# Co-designing a new clinical pathway to support families with children identified as having early-stage type 1 diabetes in Western Australia

**DOI:** 10.1007/s00125-026-06668-8

**Published:** 2026-02-03

**Authors:** Sarah K. P. Black, Alexandra Tully, Elizabeth A. Davis, Alison Roberts, Sabrina Binkowski, Leanne Cromb, Craig Taplin, Brydie-Rose Mellor, Keely Bebbington, Aveni Haynes

**Affiliations:** 1 Rio Tinto Children’s Diabetes Centre, The Kids Research Institute Australia, University of Western Australia, Nedlands, WA Australia; 2 Department of Endocrinology and Diabetes, Perth Children’s Hospital, Nedlands, WA Australia; 3 Paediatrics, UWA Medical School, the University of Western Australia, Nedlands, WA Australia

**Keywords:** Australia, Children, Co-design, Early-stage type 1 diabetes, Model of care, Pre-symptomatic type 1 diabetes, Type 1 diabetes

## Abstract

**Aims/hypothesis:**

Children with early-stage (pre-symptomatic) type 1 diabetes are currently identified primarily via research-based screening programmes in Australia. Once identified, families live with the knowledge that their child has an increased chance of developing symptomatic, lifelong, insulin-requiring type 1 diabetes but have no specific clinical pathway available to them in Western Australia (WA) for accessing tailored support or education. This project aimed to co-design a new clinical pathway to address this unmet need.

**Methods:**

Experience-based co-design (EBCD) methodology was applied, comprising three phases undertaken consecutively over 12 months. Recruitment for each phase was via open invitation with voluntary participation. Phases 1 and 2 involved facilitated community conversations and focus groups conducted separately for type 1 diabetes community members and healthcare professionals (HCPs). Data from these phases were analysed using deductive and inductive content analysis to identify key categories of information and a prototype clinical pathway was developed incorporating these. For phase 3, a combined workshop was held with all stakeholders to obtain feedback on the prototype, and refine it accordingly.

**Results:**

In phase 1, 16 community members (people living with type 1 diabetes, families whose child or children had been screened for type 1 diabetes) and 36 HCPs (doctors, nurse educators, social workers, dietitians, a mental health nurse and administrative staff from Perth Children’s Hospital) participated in separate community conversations. The following three key categories were identified: (1) the need for education; (2) the need for support and strategies for managing uncertainty; and (3) the need for information on disease-modifying therapies and access to clinical trials. In phase 2, seven community members and 11 HCPs participated in separate focus groups. The following key priorities were identified: (1) the need for access to HCPs skilled in supporting patients to manage uncertainty; (2) the need for flexibility in delivery modes (telehealth/in-person); (3) the need for early referral to relevant clinical trials; and (4) the need for reliable and up-to-date resources. In phase 3, three community members and three HCPs attended a combined workshop to provide feedback on a prototype clinical pathway and their feedback was incorporated into a final version.

**Conclusions/interpretation:**

Application of EBCD methodology enabled the development of a new clinical pathway tailored to the needs of WA families with a child living with early-stage type 1 diabetes.

**Graphical Abstract:**

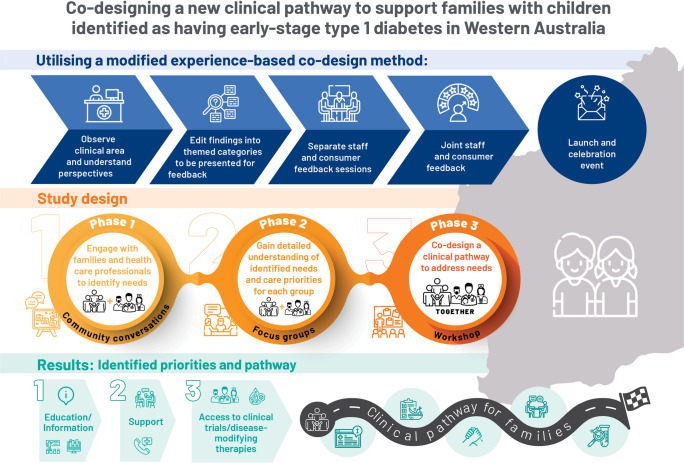

**Supplementary Information:**

The online version of this article (10.1007/s00125-026-06668-8) contains peer-reviewed but unedited supplementary material.


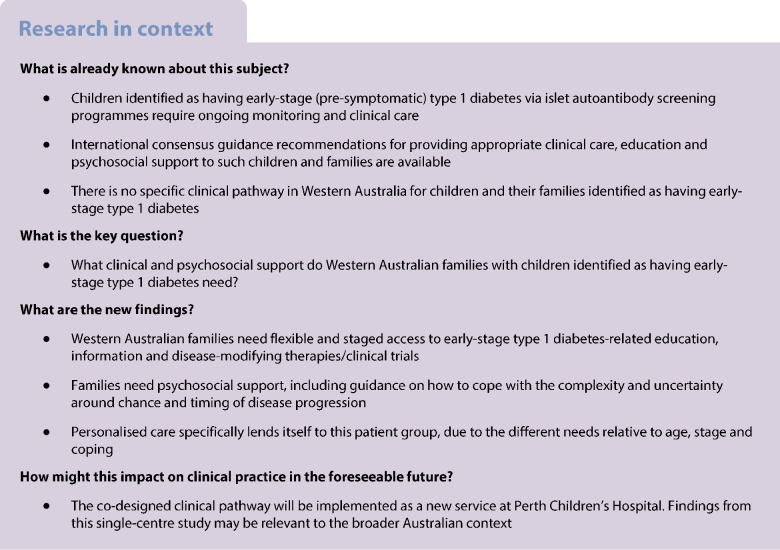


## Introduction

In Western Australia (WA), ~160 children are diagnosed with insulin-requiring type 1 diabetes annually and are managed by a multidisciplinary team at Perth Children’s Hospital [1]. While the cause of type 1 diabetes remains unknown, significant advances in the understanding of its natural history are resulting in a paradigm shift in management [2–6]. Specifically, the latent period preceding clinical onset of type 1 diabetes is now defined as two pre-symptomatic stages characterised by the presence of two or more persistent islet autoantibodies without dysglycaemia (stage 1) or the presence of asymptomatic dysglycaemia (stage 2) [7]. Stage 3 type 1 diabetes is when insulin treatment is required, and a clinical diagnosis is made [4, 8]. With disease-modifying therapies on the horizon, efforts to identify children with early (pre-symptomatic) stages of type 1 diabetes are expanding [9, 10].

Children with early-stage type 1 diabetes in Australia are primarily identified via research-based screening programmes [5, 11] and occasionally through ad hoc islet autoantibody testing conducted by general practitioners. Though general population screening is not yet available in Australia, as screening efforts expand and community awareness of early-stage type 1 diabetes grows, an increasing number of Australian children identified with early-stage type 1 diabetes is anticipated. In WA, which accounts for ~10% of the Australian population, there are currently 30 children identified with early-stage type 1 diabetes. International evidence-based recommendations [5, 6] are available, and care pathways have been established in other populations [12], including Italy, the first country to implement national general population screening [13]. However, adaptation to ensure these pathways are appropriate for the local context and acceptable to families is essential [13].

This project aimed to co-design a new clinical pathway for WA families with children identified as having early-stage type 1 diabetes, to be implemented at Perth Children’s Hospital.

## Methods

A modified experience-based co-design (EBCD) methodology was applied over a 12 month period [14, 15]. This approach to co-design promotes the collaboration of patients, carers and healthcare providers (HCPs) in the development of healthcare services. In doing so, the lived experience of service users is centred to ensure that key touchpoints in the healthcare experience are identified and service delivery can be tailored to meet their specific needs. EBCD frameworks typically consist of eight stages [16], although accepted definitions from the literature include a minimum of two phases/stages that include service users [15]. In modified EBCD, an initial phase must include using summarised service-user data to identify key touchpoints and subsequent phases must include at least one service-user participant [15].

### Participants

This project involved two key stakeholder groups: the type 1 diabetes community, including parents of children/adolescents with early-stage and clinically diagnosed type 1 diabetes (service users); and HCPs at Perth Children’s Hospital who deliver clinical care to WA children with type 1 diabetes. The research team also contributed to the co-design process and was comprised of a community involvement coordinator, research nurses, research assistants and clinician researchers, including paediatric endocrinologists and a clinical psychologist.

### Recruitment strategies

Participation by type 1 diabetes community members and HCPs was voluntary, with recruitment via open invitation. Type 1 diabetes community members eligible to participate included individuals living with type 1 diabetes (early-stage/established), individuals who were supporting a family member with type 1 diabetes, individuals who were working with young people with type 1 diabetes or individuals who had been involved in a screening programme. Type 1 community members were self-selected, responding to invitations sent via email, Perth Children’s Hospital Diabetes department newsletters and the author institutions’ social media channels. HCPs were also self-selected, responding to an email invitation to participate.

All interested participants were provided with an information sheet and given the opportunity to speak with the Children’s Diabetes Centre’s Community Involvement Coordinator (LC), or a research team member. Those who agreed to participate in the focus groups and workshop provided written informed consent.

### Ethics statement

This project was conducted in accordance with the Declaration of Helsinki [35]. The study was granted Human Research Ethics Committee (HREC) approval through the Child and Adolescent Health Service HREC in Western Australia (RGS00006772).

### Study design

#### Structural characteristics

All phases of the study were conducted in-person. For the type 1 diabetes community, phases were conducted at a community centre for families of young people living with type 1 diabetes. We offered a remote-access community conversation for regional families, or those unable to travel to the community centre in Perth, via video link. However, no families located in regional/rural areas responded to the invitation, so this did not proceed. For HCPs, phases 1 and 2 were conducted at Perth Children’s Hospital in a seminar room and phase 3 at the community centre.

Research team members shared facilitation of each phase, meeting beforehand to plan and allocate the roles of lead facilitator, co-facilitators, scribe or observer. Session guides were developed through team discussions. The team met between sessions to review data collected and methodology applied, and plan the next steps.

#### Process characteristics

**Fig. 1:**
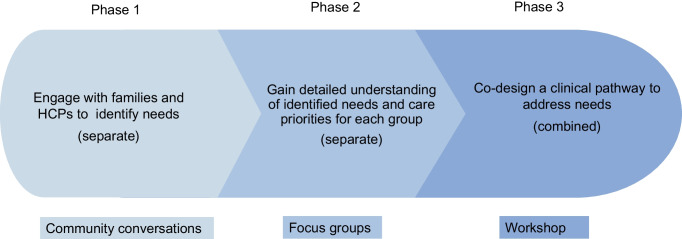
Phases of co-design study approach applied to develop a new clinical pathway to meet the needs of families with children identified as having early-stage type 1 diabetes and HCPs delivering their care

Three consecutive phases were undertaken (Fig. 1). Phase 1 involved facilitated community conversations with type 1 diabetes community members and HCPs to gain a comprehensive understanding of their needs. Phase 2 involved focus groups with these same groups to inform the development of a prototype clinical pathway. Phase 3 involved a combined workshop with participants from both stakeholder groups to obtain feedback on the prototype pathway and refine it in real-time. Data from each phase were analysed iteratively to ensure they informed materials used in the next phase. Participants’ demographic data and their relationship to the issue of early-stage type 1 diabetes were also collected.

#### Phase 1: community conversations

Participants in the type 1 diabetes community were divided into groups seated at four tables, with one research team member designated as a facilitator at each table. Participants were asked to rotate tables for each question posed, allowing varying dynamics and conversation between all participants and facilitators during the session. In addition, four research team members functioned as ‘floating’ co-facilitators and scribes.

**Fig. 2:**
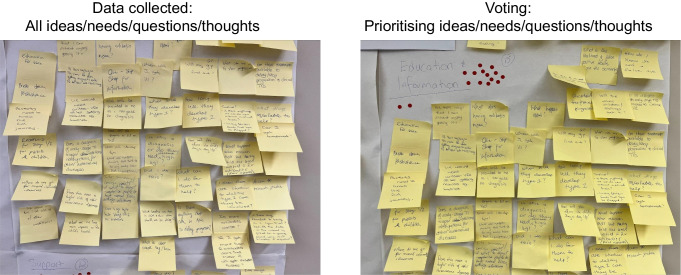
Wall display of all post-it notes used to collect data during the type 1 diabetes community conversation. Red dots indicate ideas voted as being in the top three importance for one or more participants

Questions (Table 1) were posed individually via PowerPoint, with a printed hardcopy available at each table. Ideas generated in response to questions were discussed at each table, with clarification provided by facilitators if necessary. Ideas were written on post-it notes by participants themselves, and a scribe at each table. After all questions had been addressed, all post-it notes were displayed on a noticeboard on one wall in the room, and participants were asked to prioritise them by indicating the three ideas most important to them using sticky red dots (Fig. 2). The type 1 diabetes community conversation ran for 90 min.

**Table 1:** Questions posed during the community conversation by stakeholder group

Type 1 diabetes community group	What do you need to know to help understand results from type 1 diabetes screening and being told your child has early-stage type 1 diabetes? What information and services do you think you would need and how would you want to access them?
HCP group	What is your profession? Have you heard about the stages of type 1 diabetes prior to this presentation? After our explanation do you have any initial questions or thoughts about early-stage diabetes? Do you have any initial thoughts or questions about the design of a clinical care pathway? What previous experience do you have with early-stage diabetes? How confident would you be to support these families? What education would you require to provide support for families with early-stage type 1 diabetes? What resources would you need to counsel these families? What would the clinical care for these families ideally look like? How would families best access this clinical care? Who should make up the clinical team? Is there anything you think we have missed, or do you have other questions or thoughts to add?

The HCPs’ community conversation was held during a weekly Perth Children’s Hospital diabetes departmental meeting, enabling all departmental staff to attend. All HCP participants had access to a mobile phone and the Mentimeter app [17] enabling anonymous free-text responses to be submitted during the session to each question posed (Table 1). The HCP session was also recorded via Microsoft Teams and Mentimeter responses collected for analysis. The HCPs’ community conversation ran for 60 min.

#### Phase 2: focus groups

Phase 1 participants were invited to participate in focus groups to enable deeper exploration of categories (i.e. major themes of interest) identified during their respective community conversations. The aim of the focus groups was to review these categories and discuss how they could be addressed via a new clinical pathway. To support discussion, focus group guides were structured under the headings ‘What, When, Where and How?’ to ensure ideas generated were applicable to the clinical pathway being developed (electronic supplementary material [ESM] Table 1).

The type 1 diabetes community focus group was facilitated by a member of the research team (AR) experienced in qualitative interviewing and focus group guidance. Responses to focus guide prompts (ESM Table 1) were scribed on butcher paper. This data was then transcribed by AT onto a password-protected Excel spreadsheet to enable data analysis. The session ran for 60 min.

The HCPs focus group was facilitated by a member of the research team and clinical psychologist experienced in qualitative interviewing (KB) supported by SKPB, AT, BM, AR and SB who acted as scribes and were available to assist as requested. The focus group was held at Perth Children's hospital in a seminar room with questions for HCPs presented via printed handouts (ESM Table 1). Responses were recorded by BM on butcher paper and transcribed to a password-protected Excel spreadsheet. The session lasted 60 min.

#### Prototype clinical pathway development

The coded qualitative data from both focus groups were presented to the whole research team during a face-to-face working meeting, during which a prototype clinical pathway was designed. SKPB then aligned this to recommendations identified via a review conducted by her to identify available evidence-based approaches (e.g. interventions, models of care, tools). This process included a search of peer-reviewed and grey literature (including databases such as PubMed and Google), and websites of national and international research projects undertaken within the scope of early-stage type 1 diabetes [3, 18–20]. Primary search terms used were ‘early-stage type 1 diabetes/type 1 diabetes’, ‘stage 1 type 1 diabetes’, ‘stage 2 type 1 diabetes’, ‘preclinical type 1 diabetes’ and ‘pre-symptomatic type 1 diabetes’. Secondary search terms included ‘guide’, ‘factsheet’, ‘information’, ‘booklet’, ‘video’ and ’education’. An international consensus guidance document [6], and a national adaptation of this [5], were published mid-way through this project period, from which evidence-based monitoring recommendations [5, 6] were incorporated into the draft prototype clinical pathway. Key findings and recommendations identified from the sources above were collated with the consolidated findings from phases 1 and 2 data analysis and disseminated to all participants to read prior to attending phase 3.

#### Phase 3: workshop

The workshop took place in a community centre and was attended by participants from the type 1 diabetes community, HCPs and six research team members (SKPB and AH facilitated; AR, KB, BM and AT acted as assistants and scribes). The draft prototype clinical pathway was presented to participants via PowerPoint presentation and paper copies. Fictionalised case scenarios were used to illustrate the personalised nature of the pathway according to a child’s age, number of islet autoantibodies, stage of type 1 diabetes, presence/absence of family member living with type 1 diabetes, family stress/coping and distance from Perth Children’s Hospital. Access to the clinical team and support available outside of scheduled clinic visits was also described.

Workshop participants were encouraged to provide feedback and consider local factors, such as existing resources and healthcare service models, that would impact the feasibility and sustainability of a new clinical pathway and potential barriers and facilitators to its access. Feedback was recorded on butcher paper and subsequently transcribed onto an Excel spreadsheet. Interested participants unable to attend the workshop were invited to provide feedback via email/phone or in-person to a research team member.

### Data analysis

Qualitative data from all phases were analysed iteratively, using both inductive (phase 1) and deductive (2) content analysis [21]. For phase 1, three researchers (SKPD, AT, AR) independently familiarised themselves with the data and conducted initial open coding. They then came together to discuss the codes and identify preliminary categories, reflecting broad themes within the data. For phase 2, notes taken by researchers who acted as scribes were reviewed by six members of the research team (AH, KB, SKPD, AR, AT, BM) during a 120 min in-person meeting. During this meeting, the team deductively coded the data according to the headings ‘What’, ‘When’, ‘Where’ and ‘How’ and derived subcategories of information under these headings that could be used to inform the development of the prototype clinical pathway.

SKPB drafted an initial prototype pathway informed by this analysis, incorporating evidence-based recommendations identified via a review of relevant published and grey literature. This was presented back to the whole research team for feedback and revision prior to being presented to participants during phase 3. This approach ensured member-checking, including members from both stakeholder groups and the research team, and was embedded throughout all phases, allowing for triangulation to occur.

## Results

### Participant characteristics

For phase 1, 16 type 1 diabetes community members (people living with type 1 diabetes, including three youth and families with a child [or children] screened for type 1 diabetes) and 36 HCPs (of various disciplines) participated in separate community conversations (Table 2). For phase 2, seven community members and 11 HCPs participated in separate focus groups (Tables 2, 3). Three members from each group participated in the workshop held for phase 3 (Table 2). Prior to phase 3, all participants from previous phases were provided a summary of the findings along with the draft clinical pathway and were invited to provide feedback. Two HCPs (genetic counsellor and social worker) unable to attend the workshop in person provided feedback via email.

**Table 2:** Participant characteristics by stakeholder group

Stakeholder group	No.	Attended prior phase, *n* (%)	Relationship to issue/role
Phase 1 community conversation			
T1D community	16		3 (18.8%) youth with stage 3 T1D (aged 14–18 years) 11 (68.8%) had experience screening (either selves or family member) 5 (31.3%) had a child with +ve AutoAb; one parent had one child with ES T1D and another with stage 3 T1D
HCPs	36		13 (36.1%) paediatric endocrinologists 9 (25%) diabetes nurse educators 5 (13.9%) administrative staff 3 (8.3%) dietitians 2 (5.6%) social workers 2 (5.6%) clinical research nurses 1 (2.8%) mental health nurse 1 (2.8%) nurse practitioner
Phase 2 focus group			
T1D community	7	7 (100%)	2 (28.6%) youth with stage 3 T1D (aged 14–16 years) 5 (71.4%) had a family member who had been screened for AutoAb (1 [14.3%] screened negative) 3 (42.9%) parents of children with stage 3 T1D
HCPs	11	10 (90.9%)	2 (18.2%) paediatric endocrinologists 2 (18.2%) diabetes nurse educators 1 (9.1%) genetic counsellor 1 (9.1%) nurse practitioner 1 (9.1%) social worker 1 (9.1%) clinical research nurse 1 (9.1%) mental health nurse 1 (9.1%) dietitian 1 (9.1%) administrative staff
Phase 3 workshop	6	6 (100%)	3 T1D community 1 parent with a child who has EST1D 1 parent with stage 3 T1D, child screened and negative 1 parent with 1 child with EST1D and 1 child with stage 3 T1D 3 HCPs 2 diabetes nurse educators 1 nurse practitioner

EST1D, early-stage type 1 diabetes; +ve AutoAb, positivity for islet autoantibody; T1D, type 1 diabetes

**Table 3:** Additional demographic data for focus group participants

Characteristic	Community members (*n*=7)	HCPs (*n*=11)
Mean (SD) age, years	36 (14)	
Female sex, *n* (%)	3 (42.9)	10 (90.9)
Country of birth / ethnicity	7/7 Australia / Australian	6/11 Australia / Australian 1/11 New Zealand / New Zealander 1/11 Scotland / British 1/11 England / British 1/11 England / Australian 1/11 Zimbabwe / Australian
Shortest distance from hospital centre, km	4.4	
Longest distance from hospital centre, km	37	
Have T1D themselves, *n* (%)	3 (42.9)	
Child has T1D, *n* (%)	3 (42.9)	
Partner has T1D, *n* (%)	1 (14.2)	
Family member screened for T1D, *n* (%)	5 (71.4)	
Mean (SD) length of T1D practice, years (SD)		13 (10)
Median (Q1–Q3) length of T1D practice, years		10 (5–19)

T1D, type 1 diabetes

### Prior experience of early-stage type 1 diabetes monitoring

#### Type 1 diabetes community

Of the seven participants in the type 1 diabetes community focus group, three (42.9%) had experienced islet autoantibody screening for their child. All three had attended appointments at Perth Children’s Hospital and described being unsure about whether they had found this helpful. One participant reported prior experience with continuous glucose monitoring (CGM), and all three had prior experience with self-monitoring of blood glucose (SMBG) via finger prick. The frequency of glucose monitoring ranged from daily to a few times each year.

#### HCPs

Of the 11 participants in the HCPs’ focus group, seven (63.6%) had previously discussed islet autoantibody screening with families and five (45.5%) had discussed screening results with families. The majority described the frequency of these discussions as being, ‘a few times a year’ or less. Of those who had discussed screening results with families, four (80%) had offered them a blood test (e.g. HbA_1c_), two (40%) had offered further screening for islet autoantibodies, two (40%) had offered glucose monitoring via CGM or SMBG, three (60%) had offered a follow-up appointment and four (80%) had offered education and/or resources.

### Phase 1 categories

**Fig. 3:**
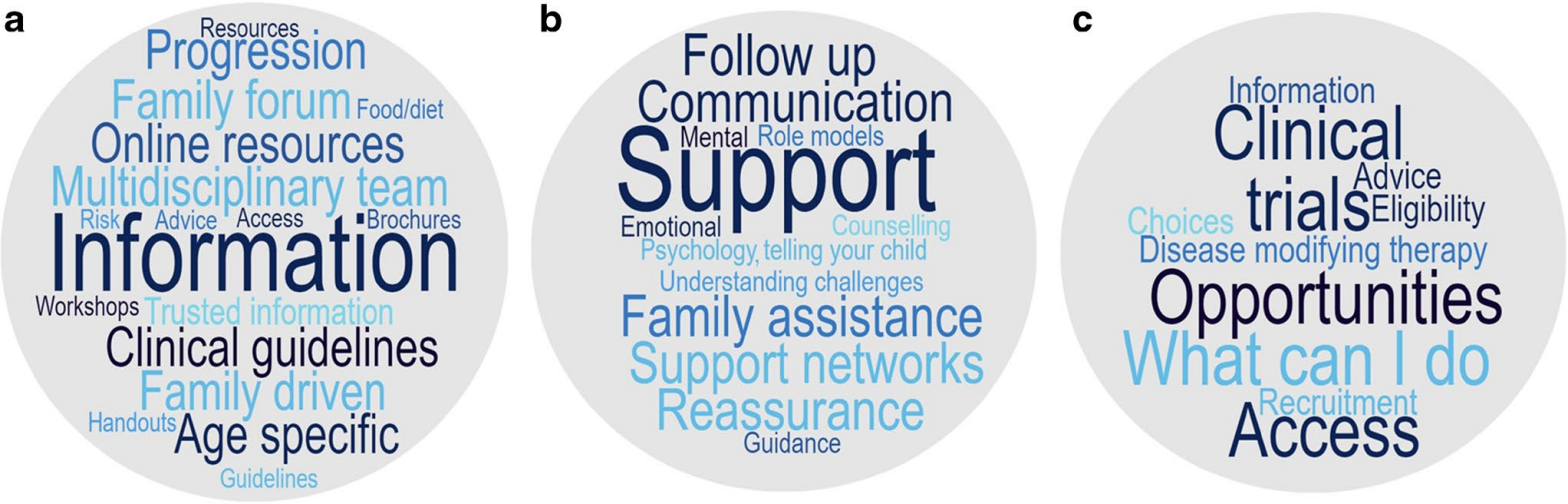
Phase 1 preliminary categories were identified from community conversations with the type 1 diabetes community and HCPs. Through iterative coding cycles, these were refined to three main categories, reflecting broad themes: (**a**) the need for education, including access to reliable evidenced-based resources and guidelines for families and HCPs; (**b**) strategies to navigate impact of uncertainty, including the need for psychosocial support; and (**c**) information and access to clinical trials and/or disease modifying therapy, as well as a plan for monitoring

Five subcategories of information were derived from the analysis of data collected during phase 1: (1) education and information; (2) support; (3) medical management; (4) technology use/access; and (5) disclosure. Through iterative coding cycles these were refined to three main categories, reflecting broad themes identified within the data (Fig. 3).

### Phase 2 categories

Participants provided more detailed insights into these three categories during the focus groups, presented below.

#### Category 1: The need for education and access to reliable information

Participants in the type 1 diabetes community group identified the need for trusted information, presented in flexible and interactive formats (e.g. combination of in-person, online and written). Education for their support network of family and friends, and primary healthcare providers was also raised. Examples of points raised included the following:

‘Resources need to be age-specific – infants, toddlers, children, adolescents’.

‘For non-diabetic relatives to understand what it is to live with the condition through education’.

Similarly, HCPs identified the need for resources to provide to families, as well as the need for access to reliable evidence-based resources and guidelines themselves.

Each group was asked to consider what information they would find most pertinent. From analysis of this data, subcategories were derived related to key topics of education (Table 4) and key priorities for educational resources identified (ESM Fig. 1).

**Table 4:** Education subcategories

Area of education identified by participants	Responses recorded by participants
Aetiology of type 1 diabetes	
Type 1 diabetes community	How did I get this? Did something I do cause this? Are they [their child] at risk of other disease?
Risk of progression	
Type 1 diabetes community	When will I get type 1 diabetes?
HCPs	Do all children with early-stage type 1 diabetes progress to clinical type 1 diabetes? We need guidelines on when stage 2 becomes stage 3
Monitoring (including financial implications)	
Type 1 diabetes community	Can you have hypos or hypers with early-stage diabetes? What do we do / where do we go if I notice my child has symptoms? Will I be on NDSS?
HCPs	What are the monitoring strategies?
Prevention and management	
Type 1 diabetes community	Are there any treatments? Do I need to change my diet, will this help delay? Will eating very low to no carbs help me last longer off insulin? How do I manage my diabetes closer to actually getting diagnosed?
Disclosure	
Type 1 diabetes community	Do I need to declare it on health and life insurance? Will we need to tell the school about it? How much do you tell others?

NDSS, Australian National Diabetes Service Scheme

#### Category 2: psychosocial support, and guidance navigating uncertainty

In the type 1 diabetes community, counselling and psychosocial support from initial detection of early-stage type 1 diabetes, with ongoing access, was emphasised as being essential. Community participants identified the need for support adjusted according to disease progression. This included support for the child and parents as well as extended family. They also identified a desire to access peer support networks. Families described needing HCPs to acknowledge the stress associated with managing uncertainty around disease progression and provide support for coping with this. The points raised included the following:

‘Is there support to the whole family to deal with the news [of having early-stage type 1 diabetes]?’‘Who can help support me emotionally, psychologically to this news [of having early-stage type 1 diabetes]?’

HCPs identified the need to be upskilled in counselling families and children to cope with uncertainty and its associated psychological impacts. They highlighted the importance of a tailored approach adapted to individuals and varying needs. Points raised included the following:

[We need to have] …‘Screening of parents and children for mental health, trauma and appropriate pathways of care, tailored approach depending on family needs’.

[We need to be upskilled in]…‘Grief and loss counselling, similar to genetic counselling. Something different to what we offer so the divide of different stages is obvious for patients’.

Both stakeholder groups emphasised positivity and hope maintenance as a priority when communicating.

#### Category 3: access to prevention and disease-modifying therapies

The type 1 diabetes community prioritised having regular updates and information on clinical trial/treatment opportunities integrated into clinical care and clear processes to monitor and reduce risk of disease progression. Points raised included the following:

‘Access to research and any trials to prevent development [of stage 3 type 1 diabetes]?’

‘Is there anything to prepare for when getting diagnosed with [early-stage] type 1 diabetes to help with transitioning [at clinical onset]?’

HCPs identified up-to-date readily accessible information and upskilling on disease-modifying therapies and clinical trials as a priority. HCPs’ suggestions for the initial clinic visit included conducting a thorough assessment of family context, including pre-existing coping strategies and current knowledge and understanding of the stages of type 1 diabetes, followed by tailored education on the child’s current stage.

Following review of the draft prototype clinical pathway, HCPs suggested the creation of a decision tree, to enable shared decision making and explain potential options for follow-up available to families. HCPs also suggested offering families a phone call soon after the initial clinic visit, along with ongoing flexible access to support between visits via phone or email, for those that needed it.

Participants from both stakeholder groups indicated a preference for visit frequency to be individualised, depending on progression and the level of psychosocial support required. They also indicated a preference for flexible service delivery to meet families’ needs (e.g. telehealth, parent-only sessions).

### Barriers to care

Some perceived barriers to establishing a new clinical pathway for children with early-stage type 1 diabetes were identified. Participants in both groups raised concerns about the impact of screening, especially on a younger child (e.g. being too young to understand such a diagnosis and the potential for psychosocial distress given the uncertainty surrounding when progression to stage 3 type 1 diabetes could occur). A question raised by a type 1 diabetes community participant was:

‘Is the impact too great for a young child who isn’t formally diagnosed?’

Other potential barriers described included the risk of physical fatigue of finger pricking or CGM wear before commencing insulin therapy, and the financial burden if families are ineligible for subsidised supplies through the Australian National Diabetes Service Scheme (NDSS). A question raised by a participant from the type 1 diabetes community was:

‘Will there be help covering the cost?’

HCP participants identified current limited access to disease-modifying therapy for early-stage type 1 diabetes in Australia as another barrier. The following points were raised:

‘Not everyone wants to know…clinically it’s needed, but some may not want to find out due to uncertainty’.

‘Potential harm in diagnosing families when more a prognosis, also what is clinical intervention?’

### Outcomes

**Fig. 4:**
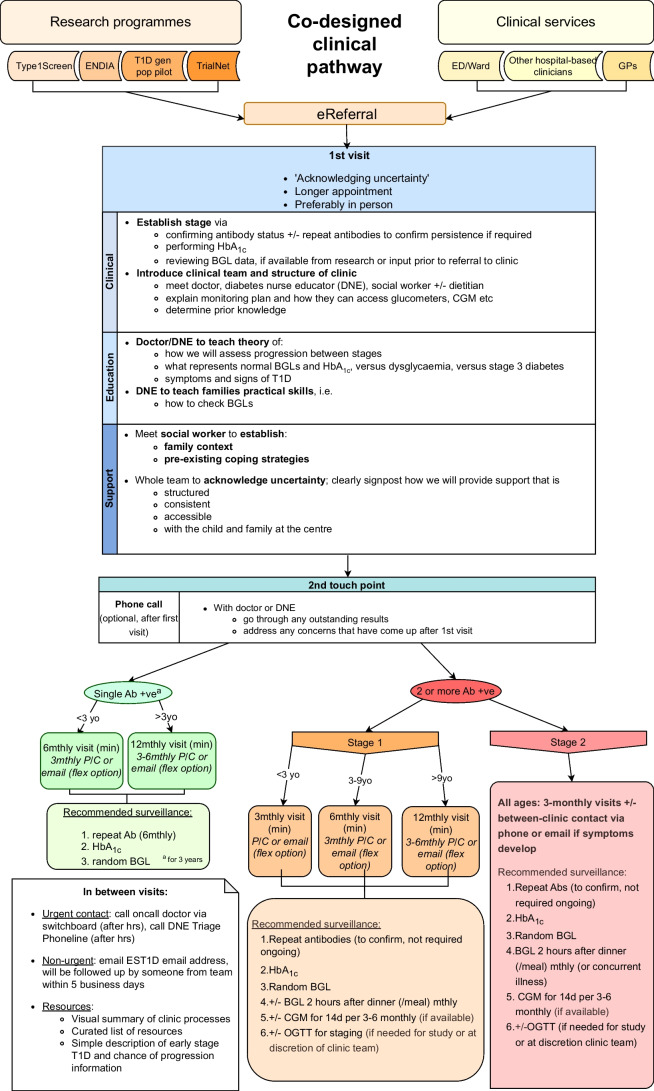
Co-designed new clinical pathway for children with early-stage type 1 diabetes in WA. Ab, antibody; Ab +ve, antibody-positive; BGL, blood glucose level; d, days; ED, emergency department; ENDIA, Environmental Determinants of Islet Autoimmunity study; EST1D, early-stage type 1 diabetes; Flex, flexible; Gen Pop, general population; GP, general practitioner; hrs, hours; min, minimum; mthly, monthly; P/C, phone call; T1D, type 1 diabetes; yo, years old

Feedback gathered at the workshop was used to revise the draft prototype clinical pathway and develop a final version (Fig. 4), the primary outcome of this project.

In addition, a short video describing the project’s rationale and methodology featuring a type 1 diabetes community participant and members of the study research team, was created [18]. A document describing the new clinical pathway, along with the video [18], was distributed to participants and the wider type 1 diabetes community in WA via email. Participants described being part of the project as a positive experience, with a member of the type 1 diabetes community making the following comment:

‘Speaking with other families, clinicians and researchers about what life looks like in the coming months, years has been a real comfort to our family... This pathway is going to be life changing for patients and their families.’

## Discussion

The co-design approach applied in this project enabled identification of needs and priorities of both type 1 diabetes community members and HCPs to inform development of a new clinical pathway for WA families with children identified as having early-stage type 1 diabetes. Notably, participants in both groups identified similar priorities from their own perspectives: (1) education and access to reliable information; (2) psychosocial support, and guidance on navigating uncertainty; and (3) strategies to delay/prevent progression, including access to disease-modifying therapies and/or clinical trials.

To our knowledge, this is the first report detailing the process and findings of applying the EBCD methodology to co-design a new clinical pathway to meet the expressed and anticipated needs of families of children with early-stage type 1 diabetes, and HCPs responsible for delivering this care. Meaningful consumer engagement in research is increasingly recognised as being essential to ensure findings are relevant and impactful [15, 16, 22]. Participants in this project included those with lived experience of type 1 diabetes or of caring for those with diagnosed type 1 diabetes or those who had participated in islet autoantibody screening (with both positive and negative antibody results), and diverse HCPs.

While participants were broadly representative of those in metropolitan and outer-metropolitan regions of WA, no participants from regional/remote areas volunteered. Specific attempts to include them (e.g. providing options for virtual attendance at the community conversation and sending feedback on the prototype pathway via email/phone) did not lead to their participation. As all children with type 1 diabetes in WA (covering a landmass of 2.5 million km^2^) are managed by the diabetes team at Perth Children’s Hospital, this is a potential limitation, as those living regionally may have differing needs. However, the new clinical pathway includes flexible healthcare delivery options, which would enable regionally located children with early-stage type 1 diabetes to have equitable access to the service.

A further limitation relates to the potential for divergence related to the pace and level of support needed by families who do or do not have prior knowledge or experience of living with a family member affected by type 1 diabetes, and whether their child was identified via at-risk vs general population screening programmes. We were unable to determine this with our current sample, although we aim to better understand this in future research.

Since this project started, an international consensus guidance document on monitoring early-stage type 1 diabetes was published [5], and an Australian adaptation made available [5]. Therefore, our new clinical pathway incorporates their recommended frequency of visits (as minimum requirements) and tools for monitoring, with additional flexible (opt-in) options for further support and varied modes of delivery, as designed by our participants [5]. Our other findings reflect many of the recommendations made surrounding education and psychosocial support; however, they add the perspective, and importantly the priorities, of families with lived experience. This project provides an example of adapting guidance documents into a region-specific model of care, while prioritising the perspectives of local stakeholders. Such adaptation is essential to ensure delivery of appropriate care that is sensitive to the local cultural and healthcare context.

Critical questions regarding how to best care for children with early-stage type 1 diabetes remain. These include the following: (1) what are the optimal approaches to, and interpretation of, glycaemic monitoring that provide sufficient accuracy and reliability to detect disease progression/need for insulin therapy while minimising burden on families? [5, 8, 23–26]; (2) how do psychosocial support needs of families differ according to a child’s age and stage? [27, 28]; and (3) by providing clinical support in early-stage type 1 diabetes do we create a ‘softer landing’ at the time of clinical onset? [29, 30]. While outside the scope of this project, we anticipate that with the implementation and evaluation of this co-designed pathway in future, we may be able to address some of these important questions. We also anticipate that by providing tailored education, resources and support, children being diagnosed with stage 3 type 1 diabetes will likely have milder symptoms and signs [12] and a significant reduction in diabetic ketoacidosis (DKA) at clinical onset, relative to those who are not identified/monitored prior to symptomatic type 1 diabetes [12, 31–34].

## Conclusions

This novel co-designed clinical pathway is the first of its kind in the rapidly evolving field of managing children with early-stage type 1 diabetes. By applying the EBCD methodology, we were able to ensure the resulting clinical pathway addresses priorities identified by both the type 1 diabetes community and HCPs. Furthermore, while aligned with current evidence-based recommendations, the resulting pathway is tailored to facilitate a fit-for-purpose pathway adapted to the local context.

## Supplementary Information

Below is the link to the electronic supplementary material.

ESM (PDF 178 KB)

## Data Availability

The data that support the findings of this study are available from the corresponding author upon reasonable request.

## Abbreviations

CGM: Continuous glucose monitoring
EBCD: Experience-based co-design
HCP: Healthcare professional
SMBG: Self-monitoring of blood glucose
WA: Western Australia
