# Supplementary material for: Co-designing a new clinical pathway to support families with children identified as having early-stage type 1 diabetes in Western Australia
Source: Diabetologia. 2026 Feb 3;69(6):1444–56. doi: 10.1007/s00125-026-06668-8 (PMC13109115; doi:10.1007/s00125-026-06668-8)
Supplement: Supplementary file 1 — ESM (PDF 178 KB) [file 125_2026_6668_MOESM1_ESM.pdf]

Supplementary Materials

Page 2	ESM Table 1: Combined focus group guide
Page 5	ESM Figure 1: Co-designed priorities for resource creation

ESM Table 1:

ESM Table 1: Focus Group Guides		
	Type 1 diabetes Community	HCP Community
What?	1) Imagine you have received a positive antibody test result. What information do you require at this point?<br>○ To understand what the result means (i.e. ensure an accurate perception of risk of developing type 1 diabetes<br>○ About disease progression?<br>○ About what life with type 1 diabetes looks like? E.g. management and clinical care<br>○ About health impacts (short and long term)?<br>2)What does support look like for you?<br>○ Professional?<br>○ Peer support?	1) Imagine you are seeing a family who has a child with a positive antibody test result. What information / resources do you need to feel confident to address this family<br>○ To understand what the result means (i.e. ensure an accurate perception of risk of developing type 1 diabetes<br>○ About disease progression?<br>○ Knowledge of current clinical trials<br>○ What else do you need<br>○ Professional? Peer support
When?	1) You've outlined some information you might wish to receive following confirmation of a positive antibody result. How soon after receiving a positive result would you like to learn about…<br>○ What your risk means<br>○ Disease progression<br>○ Living with type 1 diabetes<br>○ Health outcomes	1) Some information parents have identified as wanting to know includes:<br>○ Risk of development<br>○ Disease progression<br>○ Living with type 1 diabetes<br>○ Health outcomes<br>2) When should families start being informed of these things?

<table>
<tr>
<td></td>
<td>2) How often would you like to have contact with the care team following a positive antibody test?</td>
<td>o How often would you like to have contact with the care team following a positive antibody test?
o Do you know about current guidelines?
    o Present current expert consensus guidance document recommendations for comment [1]
o Consider Medicare rebate – will these families be eligible for multiple visits.</td>
</tr>
<tr>
<td>Where?</td>
<td>1) Do you have a preference for where you might access relevant information and support.
    o Online (e.g. self-paced education regarding early-stage and type 1 diabetes?)
    o Face-to-face meetings with a healthcare professional?
    o In the community or through the hospital?
    o Other?

2) What about ongoing contact?
    o E.g. follow up through a healthcare professional?
        ▪ GP, hospital?
        ▪ Phone check ins?</td>
<td>1) What do you think the best way would be to provide access to relevant information and support.

o Online (e.g. self-paced education regarding early-stage and type 1 diabetes?)
o Face-to-face meetings with a healthcare professional?
o In the community or through the hospital?
o Other?

2) What about ongoing contact?

    o E.g. follow up through a healthcare professional?

        ▪ GP, hospital?
        ▪ Phone check ins?</td>
</tr>
</table>

How?	1) What role do you think different professionals might have in supporting you?<br>○ Which professionals would you like access to? E.g.<br>▪ GP<br>▪ Endocrinologist<br>▪ Diabetes Educator<br>▪ Social Worker<br>▪ Psychologist/ counsellor<br>▪ Dietician<br>▪ Peer mentor	1) What role do you think different professionals might have in supporting families?<br>▪ GP<br>▪ Endocrinologist<br>▪ Diabetes Educator<br>▪ Social Worker<br>▪ Psychologist/ counsellor<br>▪ Dietician<br>▪ Peer mentor

ESM Figure 1: Co-designed priorities for resource creation

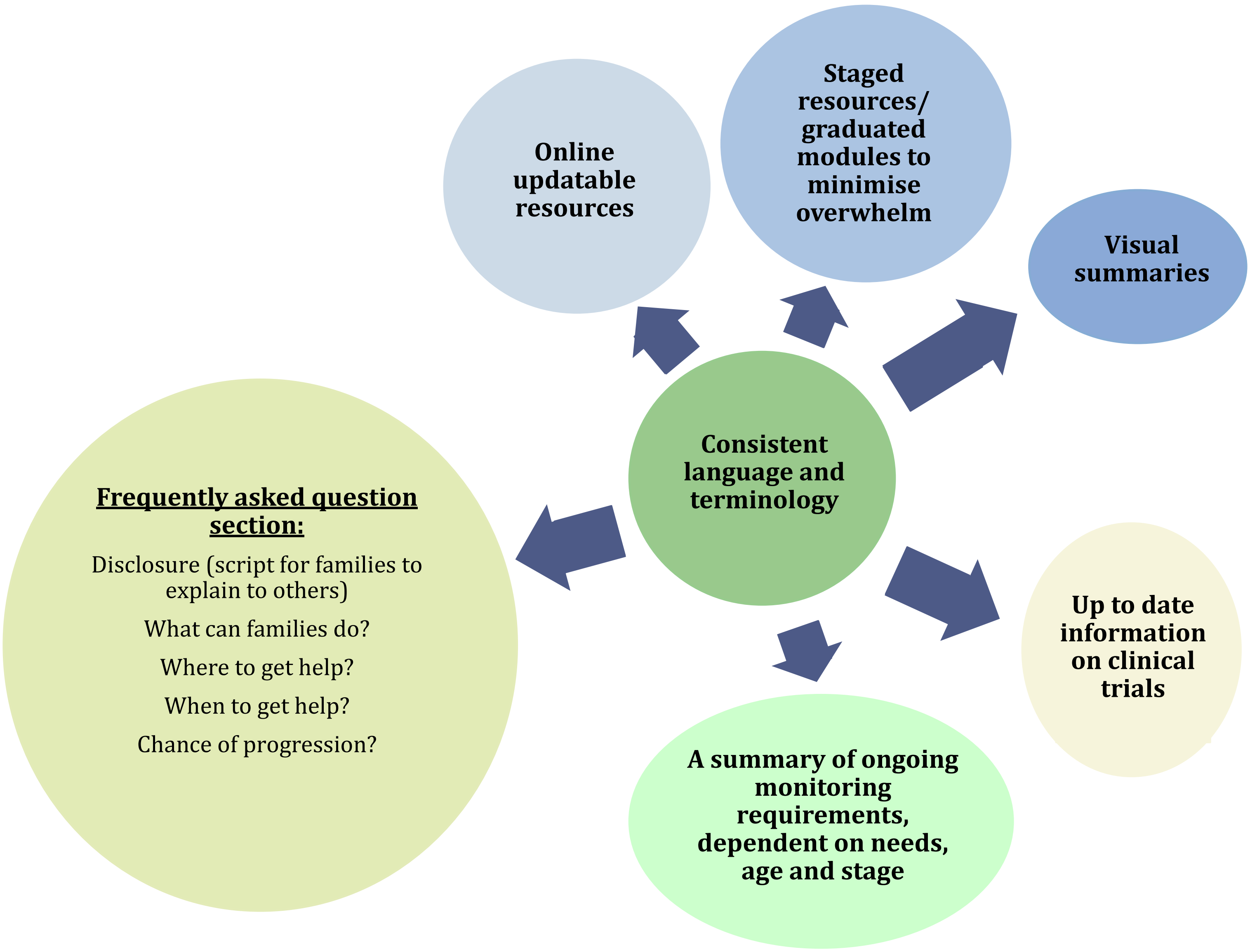

[1] Phillip M, Achenbach P, Addala A, et al. (2024) Consensus Guidance for Monitoring Individuals With Islet Autoantibody-Positive Pre-Stage 3 Type 1 Diabetes. Diabetes Care 47(8): 1276-1298. 10.2337/dci24-0042
